# Quantification of Serial Cerebral Blood Flow in Acute Stroke Using Arterial Spin Labeling

**DOI:** 10.1161/STROKEAHA.116.014707

**Published:** 2016-12-23

**Authors:** George W.J. Harston, Thomas W. Okell, Fintan Sheerin, Ursula Schulz, Phil Mathieson, Ian Reckless, Kunal Shah, Gary A. Ford, Michael A. Chappell, Peter Jezzard, James Kennedy

**Affiliations:** From the Acute Vascular Imaging Centre, Radcliffe Department of Medicine, University of Oxford, United Kingdom (G.W.J.H., J.K.); Oxford Centre for Functional MRI of the Brain, Nuffield Department of Clinical Neurosciences, University of Oxford, United Kingdom (T.W.O., M.A.C., P.J.); Department of Neuroradiology (F.S.) and Acute Stroke Service (U.S., P.M., I.R., K.S., G.A.F., J.K.), Oxford University Hospitals NHS Foundation Trust, United Kingdom; Institute of Biomedical Engineering, Department of Engineering Science, University of Oxford, United Kingdom (M.A.C.).

**Keywords:** biomarkers, cerebral infarction, magnetic resonance imaging, perfusion imaging, stroke

## Abstract

**Background and Purpose—:**

Perfusion-weighted imaging is used to select patients with acute ischemic stroke for intervention, but knowledge of cerebral perfusion can also inform the understanding of ischemic injury. Arterial spin labeling allows repeated measurement of absolute cerebral blood flow (CBF) without the need for exogenous contrast. The aim of this study was to explore the relationship between dynamic CBF and tissue outcome in the month after stroke onset.

**Methods—:**

Patients with nonlacunar ischemic stroke underwent ≤5 repeated magnetic resonance imaging scans at presentation, 2 hours, 1 day, 1 week, and 1 month. Imaging included vessel-encoded pseudocontinuous arterial spin labeling using multiple postlabeling delays to quantify CBF in gray matter regions of interest. Receiver–operator characteristic curves were used to predict tissue outcome using CBF. Repeatability was assessed in 6 healthy volunteers and compared with contralateral regions of patients. Diffusion-weighted and T2-weighted fluid attenuated inversion recovery imaging were used to define tissue outcome.

**Results—:**

Forty patients were included. In contralateral regions of patients, there was significant variation of CBF between individuals, but not between scan times (mean±SD: 53±42 mL/100 g/min). Within ischemic regions, mean CBF was lowest in ischemic core (17±23 mL/100 g/min), followed by regions of early (21±26 mL/100 g/min) and late infarct growth (25±35 mL/100 g/min; ANOVA *P*<0.0001). Between patients, there was marked overlap in presenting and serial CBF values.

**Conclusions—:**

Knowledge of perfusion dynamics partially explained tissue fate. Factors such as metabolism and tissue susceptibility are also likely to influence tissue outcome.

## Abstract 2

Supplemental Digital Content is available in the text.

In acute ischemic stroke, the relationship between regional cerebral blood flow (CBF) and tissue injury has led to the widespread acceptance of perfusion-weighted imaging to define tissue at risk of infarction.^1,2^ Perfusion-weighted imaging, using a variety of measures, has shown promise in predicting tissue outcome^3,4^ and has led to the selection of patients for clinical trials based on presenting perfusion characteristics.^1^

The majority of studies using perfusion imaging have relied on metrics derived from contrast-based magnetic resonance imaging (MRI) or computed tomography, such as time-to-peak or T_max_, which correlate with CBF.^1,5^ These biomarkers are used in clinical trials and clinical decision making,^4,6^ but their relationship to pathophysiological processes is not always clear.^7^ Arterial spin labeling (ASL) is increasingly used to measure perfusion in the context of acute ischemic stroke with the advantages that it is noninvasive, can be performed serially without repeated contrast administration without reference to renal function, and allows absolute CBF quantification.^8,9^ ASL is validated for measuring perfusion in acute stroke and compares favorably to conventional techniques.^9,10^ Multiple postlabeling delay (PLD) ASL has been less widely used in stroke-imaging studies but improves absolute quantification of CBF, even in the presence of delayed blood arrival times.^10,11^ The addition of vessel encoding attributes signal to the different feeding arteries and has the potential to further improve accuracy of CBF quantification in regions supplied by multiple arteries without compromising signal-to-noise ratio.^11^

After the advent of thrombectomy, there is a need to improve our understanding of the relationships between vessel status, reperfusion, and tissue outcome.^12^ This study aimed to investigate the relationship between changes in absolute CBF and tissue outcome in patients with acute ischemic stroke.

## Methods

### Patients and Volunteers

Six healthy volunteers were recruited and imaged under an agreed technical development protocol approved by the institution’s Research Governance Office. Patients with ischemic stroke were recruited into a prospective observational cohort study regardless of age or stroke severity under research protocols agreed by the UK National Research Ethics Service committees (refs: 12/SC/0292 and 13/SC/0362). Inclusion criteria for this analysis were the following: DWI lesion on presenting scan, presenting scan within 18 hours of symptom onset, patient or representative able to give a clear medical history and participate in the consent process, and age >18 years. Patients with a contraindication to MRI, lacunar stroke defined on DWI, or severely impaired conscious level (score >1 on question 1a of the National Institute for Health Stroke Scale) were excluded.

### Imaging

All scans were acquired using a 3.0T Siemens Verio scanner (Siemens Healthcare, Erlangen, Germany). CBF was measured using a multiple PLD vessel-encoded pseudocontinuous ASL (VEPCASL) sequence.^11^ The labeling plane was positioned ≈8 cm below the level of the circle of Willis, through the proximal V3 segment of the vertebral arteries. A single-shot echo-planar imaging readout was used, acquiring 24 slices sequentially from inferior to superior to give whole brain coverage (repetition time [TR]=4080 ms, echo time [TE]=14 ms, voxel size=3.4×3.4×4.5 mm). Volumes were acquired after a range of 6 PLDs (0.25, 0.5, 0.75, 1, 1.25, and 1.5 seconds). Vessel encoding of the 4 arteries in the labeling plane was achieved by means of 8 paired encoding cycles in a range of orientations.^11,13^ Calibration scans were acquired using identical parameters to the VEPCASL sequence but without ASL or background suppression applied, to allow absolute CBF quantification and to correct for uneven spatial sensitivity. Total acquisition time for the ASL data was 5 minutes and 55 seconds.

Retrospective motion correction was applied using the MCFLIRT tool found in the Oxford Centre for Function MRI of the Brain (FMRIB)'s Software Library (FSL).^14,15^ Calculation of the perfusion signals arriving from each artery at each PLD was achieved using a maximum a posteriori approach to the general Bayesian framework for vessel-encoded data.^13,16^ A kinetic curve was fitted to the data for each feeding artery separately to estimate CBF and bolus arrival time with a corresponding variance using a variational Bayes approach.^8,17^ The resulting vessel-specific CBF maps were summed to generate a map of the total CBF from all arteries.

Other scanning protocols included diffusion-weighted imaging (3 directions, 1.8×1.8×2.0 mm, field of view [FoV]=240 mm, 4 averages, b=0 and 1000 s/mm^2^, TR=9000 ms, TE=98 ms, 50 slices, and acquisition time=2 minutes and 53 seconds) with apparent diffusion coefficient calculation; T1-weighted structural imaging (magnetization prepared rapid acquisition gradient echo, 1.8×1.8×1.0 mm, FoV=228 mm, TR=2040 ms, TE=4.55 ms, and acquisition time=3 minutes and 58 seconds); and T2-weighted turbo spin echo fluid attenuated inversion recovery (1.9×1.9×2.0 mm, FoV=240 mm, TR=9000 ms, and TE=96 ms).

Healthy volunteers were scanned 3 times—at time 0, at 24 hours, and at 1 week—and underwent 4 repetitions of the VEPCASL sequence at each scan time. Patients were imaged at presentation, 2 hours, 24 hours, 1 week (3–9 days), and 1 month (14–42 days), whenever possible. Acute scans were defined where the presenting MRI scans were acquired within 6 hours of symptom onset. When intravenous thrombolysis was administered, the first MRI scan occurred during the infusion of alteplase.

### Image Registration

Rigid body registration using FMRIB’s Linear Image Registration Tool (FLIRT) was used for within time point registration.^14^ Nonlinear registration of structural scans was used between time points to limit potential error introduced by subacute edema.^15^ Contralateral nonischemic masks were created after registration of the perfusion deficit masks to standard (MNI152) space, before reflection and registration back to native image space.

### Definitions and Regions of Interest

In healthy volunteers, 6 regions of interest (ROIs), which were evenly distributed throughout the cerebral cortex, were derived from the Harvard-Oxford Atlas and registered into ASL image space. The regions chosen were insula, lateral occipital, middle temporal, paracingulate, postcentral, and precentral.^18^

In stroke patients, infarction at presentation was defined using semiautomated delineation of apparent diffusion coefficient below an externally validated threshold of 620×10^−^^6^ mm^2^/s.^19^ At 24 hours, infarction was manually delineated using trace DWI (b=1000 s/mm^2^) and at 1 week using T2-weighted fluid attenuated inversion recovery imaging. ROIs were defined as follows:

1. Ischemic core: within both presenting and final infarct definitions.
2. Infarct growth: within the final infarct but not within the presenting infarct, which was further divided into:
  i. Early infarct growth (infarct growth within the trace DWI infarct at 24 hours but not in the ischemic core); and
  ii. Late infarct growth (infarct growth not within the 24-hour lesion, but within the final infarct).
3. Peri-infarct: tissue that survived adjacent to the final infarct (within a dilated infarct mask [using a 3×3×3 voxel kernel], but not within the final infarct itself).

Within the ischemic core, diffusion lesion pseudonormalization was defined as regions within this ROI that had renormalized apparent diffusion coefficient values by 24 hours (>620×10^−6^ mm^2^/s). Patients were divided by reperfusion status using the Modified Treatment in Cerebral Ischemia Scale, with Modified Treatment in Cerebral Ischemia grades 2b and 3 representing reperfusion.^4,20^

Tissue segmentation of the presenting structural T1-weighted image using FMRIB’s Automated Segmentation Tool (FAST) defined gray matter partial volume estimates,^21^ which were registered into perfusion image space. Analyses were performed within gray matter masks with a partial volume estimate of ≥50%, where ASL CBF estimates are more reliable.

### Correlation With Clinical Outcomes

To investigate the relationship between perfusion dynamics and clinical recovery, changes in CBF from presentation to the 24-hour and 1-week time points within the presenting perfusion deficit were compared with changes in National Institute for Health Stroke Scale scores. Perfusion deficits were defined using a threshold of 20 mL/100 g/min to guide manual delineation of the region.

### Statistics

Means and SD of CBF within the gray matter voxels of each ROI were extracted, and patient and voxelwise means and 95% confidence intervals were calculated. Repeatability within healthy volunteers and the contralateral ROIs of patients was quantified using the coefficient of variation (SD/mean) and ANOVA. Receiver-operating characteristic curve analyses were performed to determine the utility of using VEPCASL-derived CBF at acute presentation (within 6 hours of symptom onset) in predicting infarction. Youden indices were used to estimate optimum CBF thresholds.^22^ Optimum CBF values to predict final infarction were used to estimate thresholds of ischemic core (infarct growth in reperfusers) and tissue at risk (infarct growth in nonreperfusers). To explore the effects of time of imaging on ability of CBF to predict infarction, patients were further divided into those imaged 0 to 3 and 3 to 6 hours.

## Results

Six healthy volunteers and 40 consecutive patients were prospectively enrolled and underwent serial VEPCASL imaging. Patient demographics are presented in Table.

**Table.**
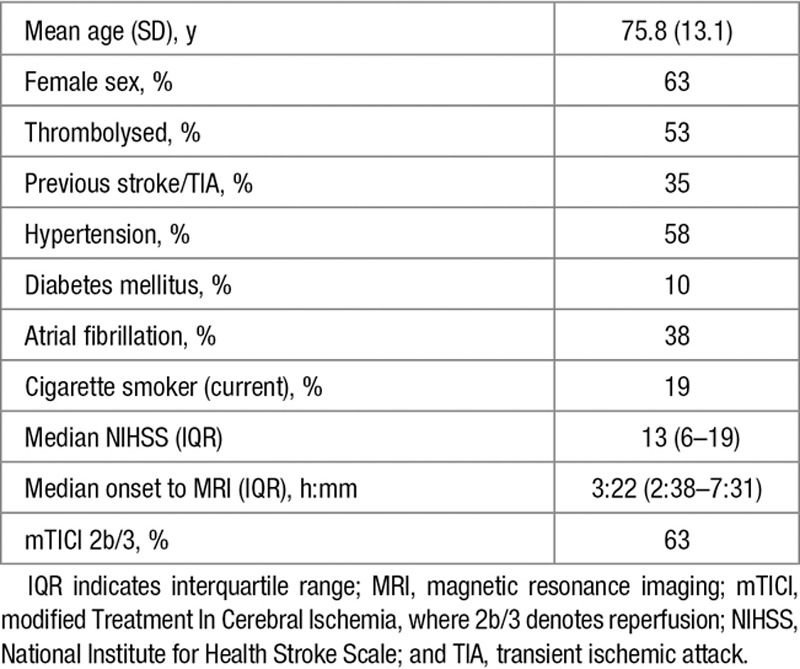
Demographic Data

### Healthy Volunteers

The average gray matter perfusion of healthy volunteers was 62±15 mL/100 g/min (mean±SD), range 40 to 78 mL/100 g/min. The coefficient of variation between the 4 repeated measures of CBF at the same scan time point was 8.5%. Between scan time points, the coefficient of variation was 9.7% (ANOVA, *P*<0.001), was 16% between individuals (*P*<0.001), and was 15% between atlas regions (*P*<0.001). Regional CBF ranged from 50±4 mL/100 g/min in the post central gyrus to 73±8 mL/100 g/min in the insula. Data are presented in Figure I in the online-only Data Supplement.

### CBF Variability in Patients

**Figure 1.**
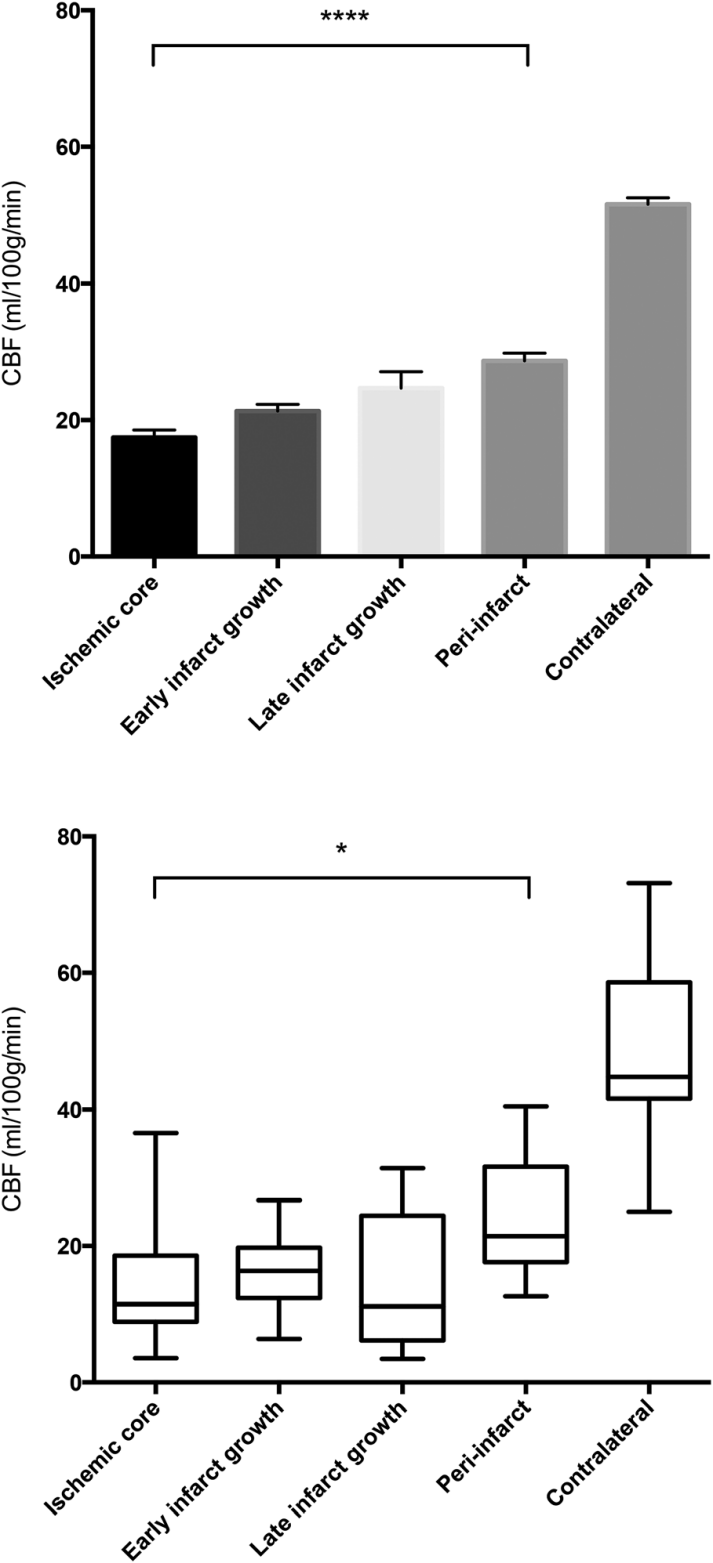
**Upper**: Voxelwise mean cerebral blood flow (CBF) in each region of interest within 6 h of symptom onset (error bars, 95% confidence interval). **Lower**: Patient-level mean CBF in each region of interest (whisker plot). ****P<0.0001; *P<0.05.

Within the contralateral ROIs of patients, the weighted mean CBF was 52±42 mL/100 g/min (Figure 1). For patients with both presenting and 24-hour scans, 2-way ANOVA demonstrated a significant effect of the individual patient on the contralateral ROI values (ANOVA, 77% of variance, *P*=0.01) but a nonsignificant effect of the day of the scan (ANOVA, 8% of variance, *P*=0.06). Dynamic CBF values from the contralateral ROIs of individual patients are presented in Figure II in the online-only Data Supplement. There was no effect of tissue-type plasminogen activator infusion or blood pressure on contralateral ROI values (*P*=0.95 and *P*=0.61).

### CBF in the Ischemic Hemisphere of Patients

At a voxel level, analysis demonstrated a graduated severity of hypoperfusion within 6 hours of stroke onset (Figure 1, **top**). Ischemic core voxels had a lower mean perfusion at presentation (mean±SD, 17±23 mL/100 g/min) than that in the regions of early and late infarct growth (21±26 and 25±35 mL/100 g/min, respectively; ANOVA, *P*<0.0001). Mean CBF in the peri-infarct ROI (29±30 mL/100 g/min) was less than that in the contralateral hemisphere (52±42 mL/100 g/min; *t* test, *P*<0.0001) but less severely hypoperfused than ROIs that infarcted (*t* test, *P*=0.002). Within the ischemic core, CBF values within regions of diffusion lesion pseudonormalization had a lower CBF than the ischemic core as a whole (8±9 mL/100 g/min; *t* test, *P*<0.0001).

At a patient level, mean CBF values showed a similar pattern to mean voxelwise ROI means (Figure 1, **bottom**; ANOVA, *P*=0.02). The variation in CBF values within and between a given type of ROI resulted in considerable overlap of values measured.

Receiver-operating characteristic curve analysis of CBF generated an area under the curve (AUC) of 0.71 for predicting final infarct using all patients scanned within 6 hours. Using only those patients with demonstrated reperfusion to predict the ischemic core generated a CBF threshold, defined by the Youden analysis, of 22 mL/100 g/min and AUC of 0.75. The optimum threshold for final infarction in nonreperfusers was 25 mL/100 g/min with an AUC of 0.72 (Figure III in the online-only Data Supplement). Subgroups of patients who were imaged at 0 to 3 and 3 to 6 hours had similar optimum thresholds and AUCs to the larger 0 to 6 hours cohort, except those who reperfused and were imaged before 3 hours, when the optimum threshold was 14 mL/100 g/min with an AUC of 0.76 (Table I in the online-only Data Supplement).

### Serial CBF Measures

**Figure 2.**
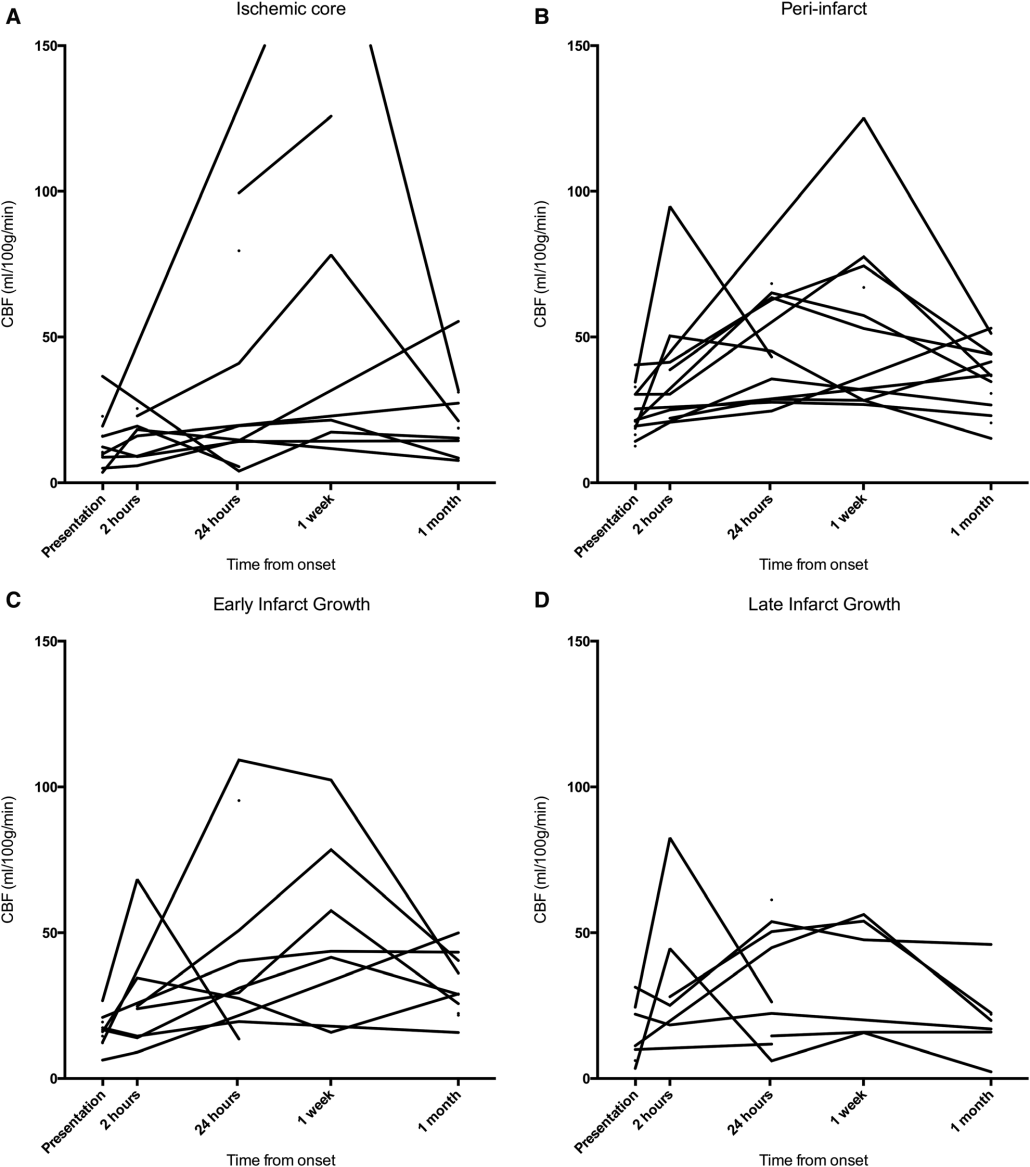
Serial cerebral blood flow (CBF) values from different regions of interest at a patient level (presentation within 6 h of symptom onset).

**Figure 3.**
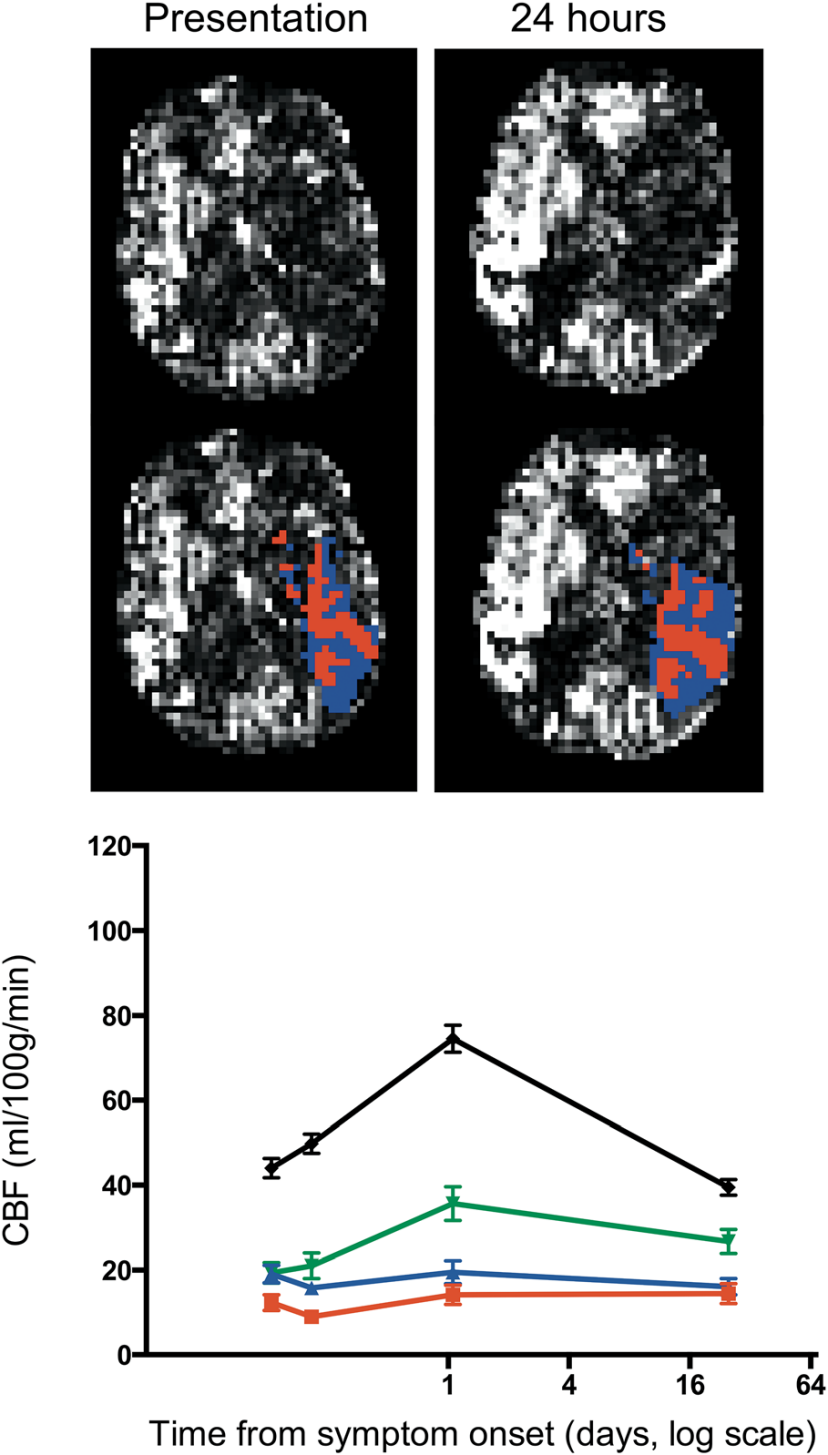
**Upper**: Example cerebral blood flow (CBF) maps from a patient without reperfusion at presentation and 24 h, with superimposed regions of interest below (red, ischemic core and blue, infarct growth). **Lower**: Absolute CBF quantification in the 4 regions of interest (red, ischemic core; blue, infarct growth; green, peri-infarct; and black, contralateral).

**Figure 4.**
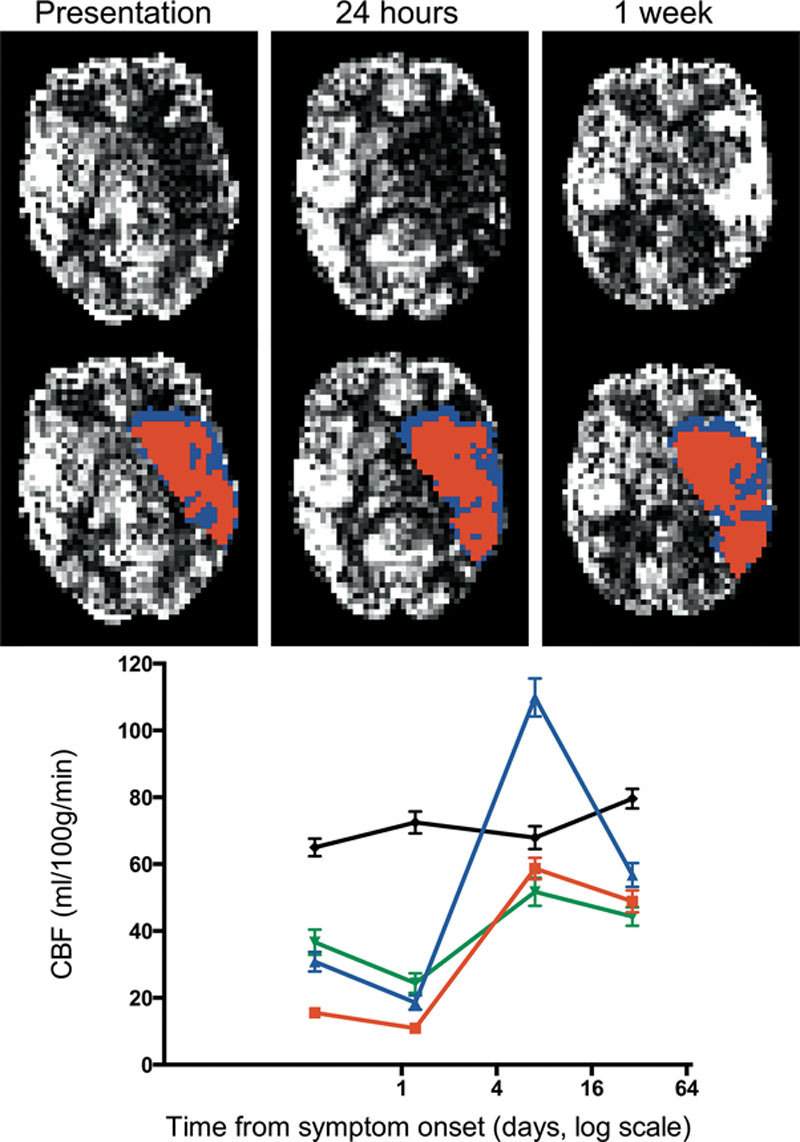
**Upper**: Example cerebral blood flow (CBF) maps from a patient with delayed reperfusion and localized hyperemia at presentation, 24 h, and 1 wk, with superimposed regions of interest below (red, ischemic core and blue, infarct growth). **Lower**: Absolute CBF quantification in the 4 regions of interest (red, ischemic core; blue, infarct growth; green, peri-infarct; and black, contralateral).

There was marked heterogeneity in the patterns of perfusion within identically defined ROIs between patients (Figure 2). Both sustained ischemia and reperfusion to varying degrees were seen in the ischemic core and early and late infarct growth ROIs. The only uniform pattern was seen in peri-infarct ROIs, which by definition survived, where all patients demonstrated a CBF value of >20 mL/100 g/min by 24 hours. Examples of the dynamic changes in CBF within individual patients with differing degrees of reperfusion, together with example CBF data, can be seen in Figures 3 through 5. Where hyperemia was seen post reperfusion at 24 hours and 1 week, the CBF had returned to low or normal levels in all patient ROIs at 1 month (Figures 2 and 4).

**Figure 5.**
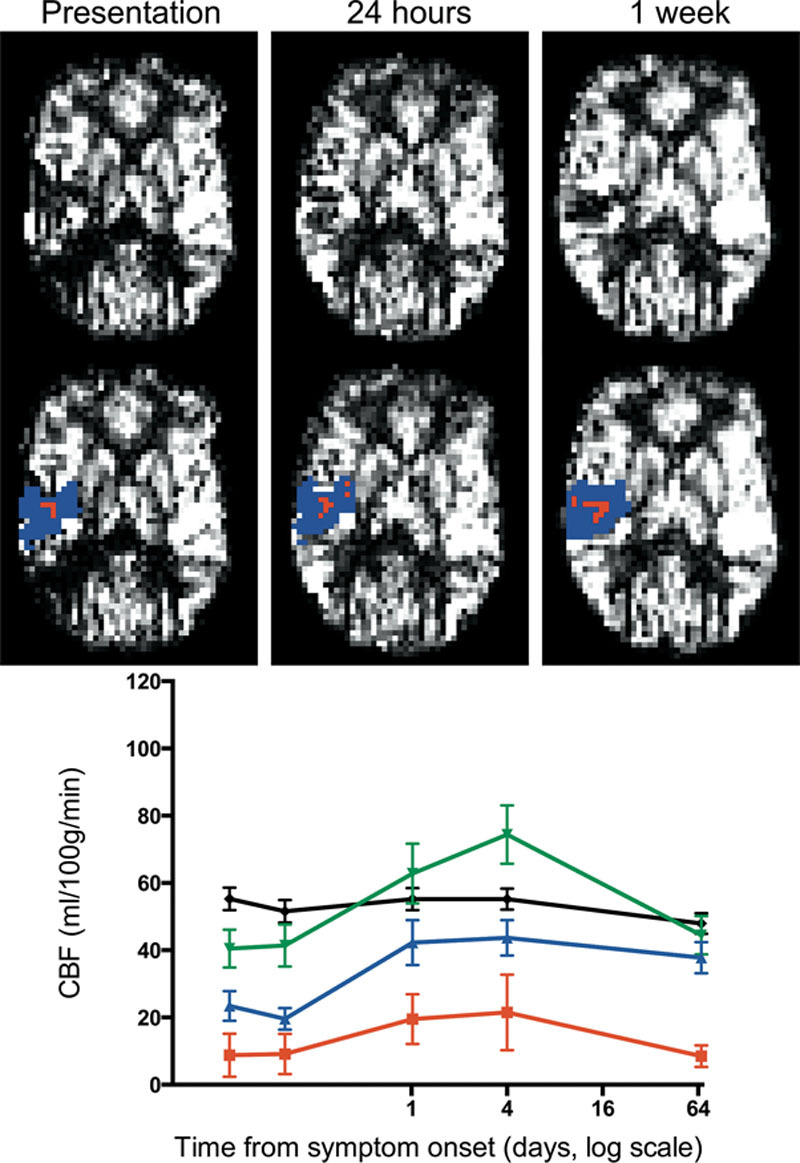
**Upper**: Example cerebral blood flow (CBF) maps from a patient with partial reperfusion at presentation, 24 h, and 1 wk, with superimposed regions of interest below (red, ischemic core and blue, infarct growth). **Lower**: Absolute CBF quantification in the 4 regions of interest (red, ischemic core; blue, infarct growth; green, peri-infarct; black, contralateral).

### Correlation With Clinical Outcomes

Increases in CBF within the presenting perfusion deficit by 1 week predicted a reduction in National Institute for Health Stroke Scale score (*r*^2^=54%; *P*=0.03). No correlation was seen between presentation and 24 hours (*P*=0.9).

## Discussion

Knowledge of perfusion dynamics partially explained tissue outcome in this cohort of patients with acute ischemic stroke. At presentation, mean voxelwise CBF measured using multiple PLD VEPCASL was consistent with tissue outcome in the ROIs of patients. The absolute CBF values were comparable to those derived using contrast and radiolabeled techniques in both healthy individuals and patients with stroke.^23–26^ A biologically plausible pattern was observed: ROIs that underwent infarction earliest had a lower presenting CBF than those of later infarct growth. For example, regions of ischemic core had the lowest blood flow at presentation, but within this ROI, the voxels that underwent diffusion pseudonormalization had lower still presenting CBF values. This phenomenon is thought to represent facilitated diffusion from early vasogenic edema and a more severe injury,^27,28^ and the CBF in this ROI was similar to cited thresholds of membrane failure (8 mL/100 g/min).^29^

Receiver-operating characteristic curve analyses at presentation demonstrated that the ability of CBF to predict final infarction was only fair. CBF thresholds were similar to those estimated using contrast techniques and with equivalent AUCs.^30,31^ There was only small improvement in the AUCs when those with and without reperfusion were considered separately, implying that knowledge of presenting CBF and a final reperfusion status is not sufficient to predict tissue outcome. Subdividing patients into those imaged before and after 3 hours did not improve CBF prediction of infarction but did identify a lower threshold for defining ischemic core in patients imaged before 3 hours. This is consistent with preclinical data describing increasing CBF thresholds for infarction over time.^32^ Factors that confound using a single CBF measurement to predict tissue fate include duration and degree of previous hypoperfusion, dynamics of reperfusion, tissue type, previous exposure to ischemia, ischemia–reperfusion injury, and individual susceptibility to hypoperfusion,^33–36^ along with measurement errors such as residual motion artifacts and insensitivity to delayed blood arrival.

At a patient level, this finding was borne out by the marked overlap of presenting CBF values in different ROIs, demonstrated in Figure 1. A similar pattern of mean CBF values to that of the voxelwise analysis was seen across the different tissue outcomes. Again, a single CBF measurement to predict tissue outcome at presentation in an individual is not sufficient, even with knowledge of final perfusion status.

The predominant source of variability in the measurement of CBF is the variation seen between individuals, as opposed to temporal variation within individuals and other factors including noise. This individual variation may reflect differences in age,^26^ hypertension,^37^ and burden of preexisting cerebrovascular disease.^26^ This has implications when quantifying CBF values relative to the contralateral hemisphere. Alternatively, systematic measurement bias that remained consistent across time points, such as anatomic factors affecting labeling efficiency, tendency to move in the scanner, and variations in vascular anatomy, may have explained some of the observed interindividual variation. Although there was some variation over time in the contralateral ROIs of patients, CBF did not vary systematically between the presenting scan and at 24 hours, which might have been expected if diaschisis had a significant effect.^38,39^

Analysis in healthy volunteers showed small, but statistically significant, variations in CBF between individuals, day of scan, and region of the brain, comparable to other work.^40^ These variations in healthy CBF values may explain some of the variations seen in patients. However, the magnitude of the variation in healthy volunteers was low and well within the recommended limits for variation in ASL studies (20%),^41^ and less than that seen in contrast-based perfusion MRI.^42^

Serial perfusion data within individuals emphasized that perfusion dynamics are consistent with tissue outcome in some, but not all, patients. Data in Figure 2 from identical ROIs across patients show that reperfusion characteristics are heterogeneous. In the peri-infarct ROIs, which represent tissue that survives, CBF consistently recovers by 24 hours to a greater level than the thresholds for infarction. However, reperfusion is not sufficient for survival of ischemic tissue outside the ischemic core, demonstrated by the reperfusion observed in regions of infarct growth. It is likely that only early reperfusion will allow tissue survival, but even this may not be sufficient for recovery. Delayed tissue injury despite recanalization is well described,^43^ and measuring serial absolute tissue perfusion may help to differentiate no-reflow phenomena from ischemia–reperfusion injury.^44^

Increases in CBF within the presenting perfusion deficit correlated with clinical recovery at 1 week but not at 24 hours. The lack of correlation at 24 hours may be because the study is too small for these patient-level analyses. Additionally, the correlation at 1 week may have been emphasized by hyperemia in reperfusers at 1 week (Figure 4).

All ASL studies are limited by the challenges of measuring late arriving blood and white matter perfusion. Even after using multiple PLD ASL techniques, it is not possible to distinguish late arriving blood from voxels with minimal CBF. Combined with effects of partial volume contamination from white matter and CSF, this may lead to systematical underestimation of CBF. Other limitations include data loss in this study, a combination of motion artifact and loss to follow-up, which is more likely to occur in severe stroke syndromes. Advances using prospective motion correction, improved acquisition techniques, and shorter imaging times may mitigate some of these sources of error in the future.

## Conclusions

This study explores the relationship between tissue-level perfusion and highly characterized tissue outcome in patients with acute ischemic stroke. Without using exogenous contrast, CBF values were derived that were consistent with other more invasive techniques. The ability to acquire serial data highlighted the heterogeneity of perfusion characteristics between individuals and the need for complementary information, including tissue susceptibility and metabolism, to fully understand tissue fate in acute stroke.

## Acknowledgments

We wish to acknowledge the facilities provided by the Oxford Acute Vascular Imaging Centre and the staff of the Oxford Acute Stroke Programme.

## Sources of Funding

This study was supported by the National Institute for Health Research Oxford Biomedical Research Centre Programme, the National Institute for Health Research Clinical Research Network, the Dunhill Medical Trust (grant number: OSRP1/1006), the Royal Academy of Engineering, and the Centre of Excellence for Personalized Healthcare funded by the Wellcome Trust and Engineering and Physical Sciences Research Council (grant number WT088877/Z/09/Z).

## Disclosures

Dr Ford has received grant support from the National Institute of Health Research. Dr Ford has also received personal remuneration for advisory work from Astra Zeneca, Daiichi Sankyo, and Pfizer. Dr Ford has received lecture fees from Medtronic. Dr Chappell has received royalties for commercial licenses from the FMRIB software library. Dr Chappell and Dr Okell have received royalties from commercial licenses from Siemens from the vessel-encoding image-processing software. The other authors report no conflicts.

## Supplementary Material
