# Supplementary material for: Quantification of Serial Cerebral Blood Flow in Acute Stroke Using Arterial Spin Labeling
Source: Stroke. 2016 Dec 23;48(1):123–30. doi: 10.1161/STROKEAHA.116.014707 (PMC5175999; doi:10.1161/STROKEAHA.116.014707)
Supplement: Supplementary file 1 [file str-48-123-s001.pdf]

ONLINE SUPPLEMENT

Contents

1. Supplementary Table
2. Supplementary Figures

1. Supplementary Table

Time from onset to MRI	0-3 hours		3-6 hours	
Reperfusion status	Reperfusion	No reperfusion	Reperfusion	No reperfusion
Optimum CBF threshold (ml/100g/min)	14	25	27	25
Area under the curve	0.76	0.77	0.74	0.65

Table I – Receiver operating characteristic curve analyses for predicting final infarction in patients who reperfuse and those who do not reperfuse.

2. Supplementary Figures

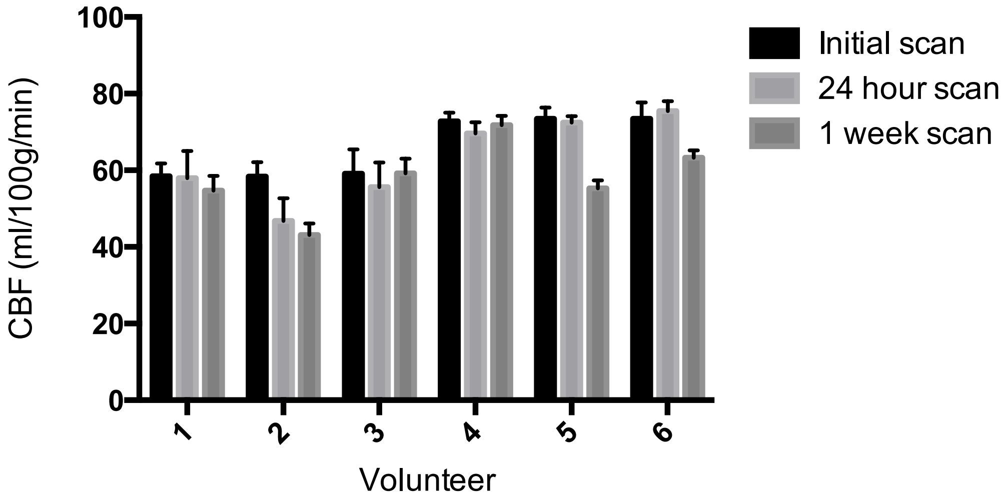

Supplementary Figure I - Cerebral blood flow (CBF, mean and standard deviation) in 6 healthy volunteers. Each bar represents four repeat measurements at one of three scan times. ANOVA showed significant variation between both individuals and scan times ($p<0.0001$).

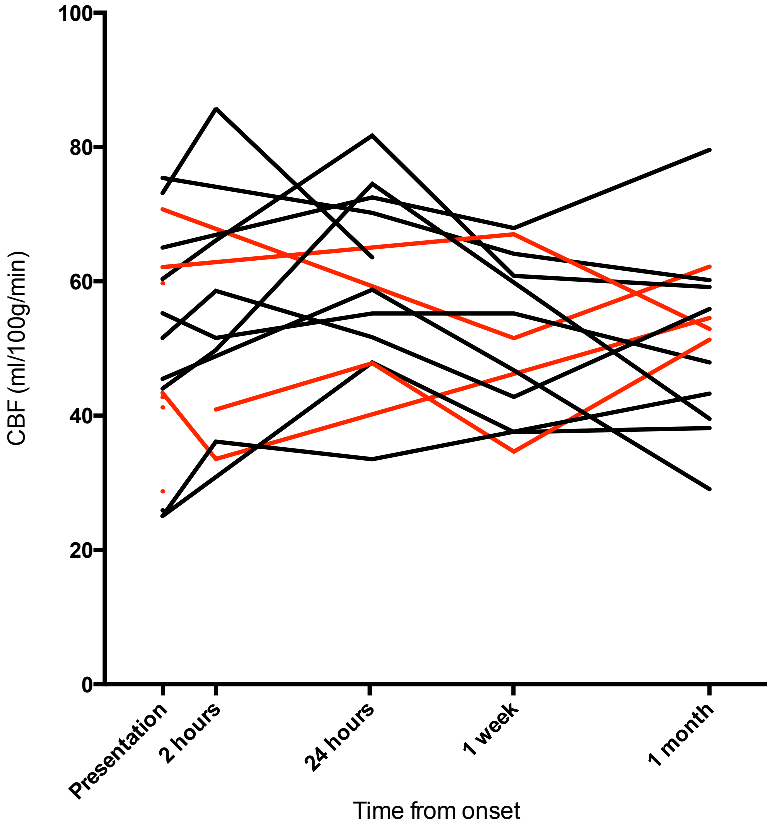

Supplementary Figure II – Contralateral cerebral blood flow (CBF) values over time in individual patients. Red lines = patients infused with tPA acutely.

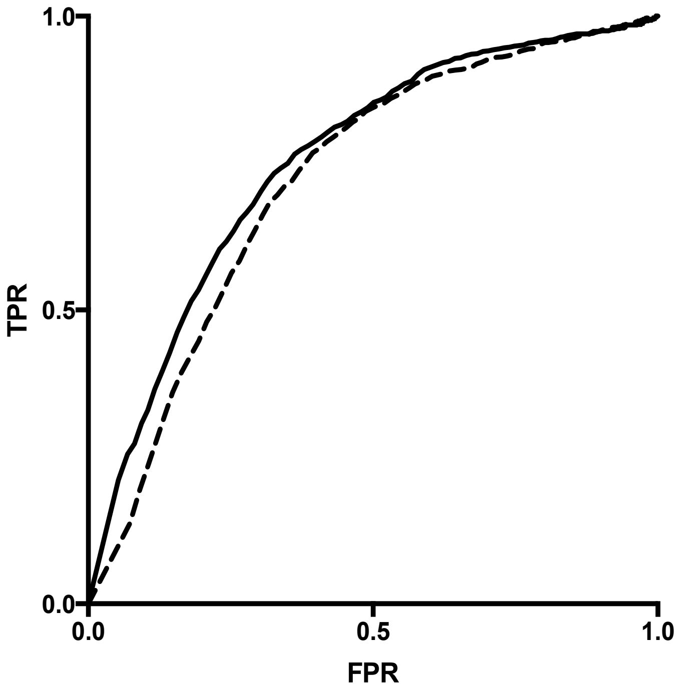

Supplementary Figure III - Receiver operating characteristics (ROC) curves for predicting final infarct within 6 hours using presenting CBF in patients who reperfuse (solid line), and do not reperfuse (intermittent line). TPR = true positive rate; FPR = false positive rate.
